# Advancing Efforts to Achieve Health Equity: Equity Metrics for Health Impact Assessment Practice

**DOI:** 10.3390/ijerph111111054

**Published:** 2014-10-24

**Authors:** Jonathan Heller, Marjory L. Givens, Tina K. Yuen, Solange Gould, Maria Benkhalti Jandu, Emily Bourcier, Tim Choi

**Affiliations:** 1 Human Impact Partners, 304 12th Street, #2B, Oakland, CA 94607, USA; 2 Department of Population Health Sciences, School of Medicine and Public Health, University of Wisconsin, Madison, WI 53726, USA; E-Mail: mgivens@wisc.edu; 3 National Association of County and City Health Officials, 1100 17th Street NW, Seventh Floor, Washington, DC 20036, USA; E-Mail: tyuen@naccho.org; 4 School of Public Health, University of California, Berkeley, 50 University Hall #7360, Berkeley, CA 94720, USA; E-Mail: solange.g@icloud.com; 5 Institute of Population Health, University of Ottawa, 1 Stewart Street, Room 300, Ottawa, ON K1N 6N5, Canada; E-Mail: maria.benkhalti.jandu@uottawa.ca; 6 Center for Community Health and Evaluation, Group Health Research Institute, 1730 Minor Avenue, Suite 1600, Seattle, WA 98101, USA; E-Mail: bourcier.e@ghc.org; 7 Solano County Health and Social Services Department, 275 Beck Avenue, MS 5-240, Fairfield, CA 94533, USA; E-Mail: tmchoi@solanocounty.com

**Keywords:** Health Impact Assessment, equity, methods, metrics

## Abstract

Equity is a core value of Health Impact Assessment (HIA). Many compelling moral, economic, and health arguments exist for prioritizing and incorporating equity considerations in HIA practice. Decision-makers, stakeholders, and HIA practitioners see the value of HIAs in uncovering the impacts of policy and planning decisions on various population subgroups, developing and prioritizing specific actions that promote or protect health equity, and using the process to empower marginalized communities. There have been several HIA frameworks developed to guide the inclusion of equity considerations. However, the field lacks clear indicators for measuring whether an HIA advanced equity. This article describes the development of a set of equity metrics that aim to guide and evaluate progress toward equity in HIA practice. These metrics also intend to further push the field to deepen its practice and commitment to equity in each phase of an HIA. Over the course of a year, the Society of Practitioners of Health Impact Assessment (SOPHIA) Equity Working Group took part in a consensus process to develop these process and outcome metrics. The metrics were piloted, reviewed, and refined based on feedback from reviewers. The Equity Metrics are comprised of 23 measures of equity organized into four outcomes: (1) the HIA process and products focused on equity; (2) the HIA process built the capacity and ability of communities facing health inequities to engage in future HIAs and in decision-making more generally; (3) the HIA resulted in a shift in power benefiting communities facing inequities; and (4) the HIA contributed to changes that reduced health inequities and inequities in the social and environmental determinants of health. The metrics are comprised of a measurement scale, examples of high scoring activities, potential data sources, and example interview questions to gather data and guide evaluators on scoring each metric.

## 1. Introduction

Over the last several decades, public health evidence has led to an increased focus on the role of social and environmental determinants of health—that is the context in which we live, work, and play—in shaping health and health equity. Within the context of Health in All Policies, Health Impact Assessment (HIA) has emerged worldwide as a practical and useful tool for addressing the determinants of health. HIA provides stakeholders and decision-makers with information about how proposed policies, plans, programs, and projects are likely to affect the determinants of health and offers recommendations that mitigate negative and augment positive effects. The process of conducting an HIA can build or strengthen relationships between stakeholders and can engage and empower populations who are likely to be affected by a proposal and who may already face poor health outcomes and marginalization. HIAs often recognize the lived experience of those populations as important evidence.

One of the primary reasons public health practitioners have focused on the determinants of health is to reduce inequities [1], such as the stark and unjust differences in social, economic, and material conditions between populations [2]. Fundamental to HIA practice is a focus on addressing the determinants of health to advance equity, and the intrinsically linked principles of democracy. Guiding documents for the field clearly articulate equity and democracy as two of the underlying values of HIA [3,4]:

(1) Equity—“emphasizing that HIA is not only interested in the aggregate impact of the assessed policy on the health of a population but also on the distribution of the impact within the population, in terms of gender, age, ethnic background and socio-economic status, [or other attributes];”
(2) Democracy—“emphasizing the right of people to participate in a transparent process for the formulation, implementation and evaluation of policies that affect their life, both directly and through the elected political decision-makers.”

Recent studies have examined the incorporation of equity into HIA practice and reported that there are opportunities for improvement [5,6]. To translate the inclusion of equity and democracy in practice, efforts have been made to provide guidance and tools. These include, for example, the development of equity-focused HIA in Australia [7,8], the inclusion of equity as an important motivation for stakeholder participation in HIA [9], and a primer entitled “Promoting Equity Through the Practice of Health Impact Assessment” [10].

Adding to the practical application of these resources, a set of clear metrics for evaluating the degree to which an HIA successfully incorporated equity could encourage HIA practitioners to take a more intentional and thorough approach to addressing equity. Developing such metrics could also serve as a resource for evaluators, equity advocates, and others interested in assessing the progress toward equity within and across HIAs conducted. With this in mind, over the course of a year, the Society of Practitioners of Health Impact Assessment (SOPHIA) Equity Working Group collaborated in a consensus process to develop a set of process and outcome metrics and an assessment tool, henceforth referred to as the Equity Metrics, for assessing and promoting equity through HIA.

This article describes the development and testing of the Equity Metrics that aim to compare the progress toward equity by HIA practitioners within and across HIAs. These metrics provide a more detailed approach to the “Minimum Elements and Practice Standards for Health Impact Assessment” [11] regarding the incorporation of equity into HIA practice, and provide a practical resource to guide assessments grounded in the underlying values of HIA. The use of these metrics may promote a greater systematic and explicit inclusion of equity considerations within HIA and within the broader field of public health.

The Equity Metrics are organized into four outcomes:

(1) The HIA process and products focused on equity.
(2) The HIA process built the capacity and ability of communities facing health inequities to engage in future HIAs and in decision-making more generally.
(3) The HIA resulted in a shift in power benefiting communities facing inequities.
(4) The HIA contributed to changes that reduced health inequities and inequities in the social and environmental determinants of health.

The first two outcomes, when achieved, can contribute to the final two outcomes, which can take place over a longer time period after the HIA has been completed or the decision has been finalized.

## 2. Experimental Section

The metrics were initiated by the Equity Working Group at the 2013 HIA of the Americas Workshop in Oakland, California. The Workshop is a meeting of HIA practitioners held every 18 months to discuss the state of the field in the Americas and to plan steps for future advancements. Multiple working groups were convened as part of the Workshop. Workshop participants self-selected into the Equity Working Group based on interest and expertise. The members ranged in affiliations: a local health department, academia, national non-profits, and an evaluation firm. Members also varied in experience with HIA, from new practitioners to practitioners with many years of experience. Each of the authors brought to bear their own expertise on and experiences with evaluation, equity, community engagement, and HIA practice.

During the Workshop, the Equity Working Group discussed the need for the HIA field to improve and increase the inclusion and consideration of equity in HIA and decision-making. The participants identified several potential joint projects and prioritized the development of a set of metrics that would help bridge theory and practice, as well as evaluate whether and how equity has been advanced through HIA. After the Workshop, the authors continued to meet monthly over the course of a year to discuss the development of the metrics, and collaborated over the framing, content, scope, and format of the draft metrics. The metrics were developed iteratively in a consensus process over the course of multiple discussions.

After the development of a draft set of the Equity Metrics, HIA practitioners were solicited to pilot test them. A convenience sample of ten practitioners with an interest in advancing equity was recruited via email from a pool of known contacts. Some of these practitioners shared the email with other practitioners who also provided input. Additionally, an online survey, comprised of 11 questions, was developed to evaluate application of the tool and collect feedback for possible improvements. Six HIA practitioners provided feedback on the use of the equity metrics to evaluate at least eight completed HIAs. Five practitioners completed the survey. Those who piloted the metrics represented HIA practitioners in the United States and Australia, and used the metrics to assess HIAs on a range of decisions, including transportation and criminal justice policies, redevelopment projects, or land-use plans. Results were summarized using descriptive statistics from the survey and qualitative themes from the open-ended responses. This feedback was utilized to revise the Equity Metrics, as described in the results and discussion section.

## 3. Results and Discussion

Five survey respondents provided the following insight into the use of the Equity Metrics.

(1) Three rated the level of difficulty as “very easy” or “just right”; two felt that the tool was “somewhat difficult” to use.
(2) Four respondents either “strongly agree” or “agree” with the statement that the metrics were useful for evaluating equity in HIA and/or proactively thinking about how to include equity considerations in HIA. One disagreed with the statement.
(3) Two respondents sought outside input from other team members or stakeholders when completing the metrics, and the same respondents also stated that they felt that too much time was involved in using the metrics.
(4) On average, it took respondents 1.9 h to evaluate an HIA using the metrics.
(5) our stated that they would use the metrics again. Three would recommend this tool to other HIA practitioners.

The open-ended feedback provided by the reviewers was varied. Several challenges to using the Equity Metrics were provided.

(1) Several projects were not able to score the more distal metrics, outcomes 3 and 4, which generally require more time to measure following the completion of an HIA.
(2) One reviewer felt that the metrics’ outcomes were multifactorial and unclear, and the tool privileged procedural approaches (community capacity building, consultation, and engagement).
(3) Another reviewer shared that the metrics were very specific to a comprehensive HIA, rather than other forms of HIA or Health in All Policies (HiAP) approaches.

Respondents also reported on strengths of the Equity Metrics, such as the usefulness of examples of high scoring activities to fully understand the metrics. One pilot tester reported that using the metrics had allowed them to understand and learn more about the two HIAs they participated in. The Equity Metrics also served as a reminder that community engagement can be difficult and may require a significant investment of time and energy. Respondents also commented on how the metrics could encourage practitioners to increase equity considerations in future HIA practice.

An important theme that emerged from the feedback is that respondents do not engage with community members to the degree that these metrics assume. Respondents noted several reasons for this. Often, HIA is conducted within HiAP contexts such as within planning departments, or other governmental agencies. Sometimes these agencies do not have a mandate for equity, and approach communities for consultation rather than engagement and empowerment. One respondent said that sometimes the Terms of Reference, or rules governing the HIA, specifically do not allow for community engagement. Another respondent stated that in their context, HIAs are not released to communities unless the community is actively involved in the HIA, and it usually is not. One respondent stated that in an HiAP approach, practitioners “often intentionally do not go out into the community because ‘policy’ is an internal matter for government and to bring in community is not part of the policy process. I think there is room for these types of assessments even though they are not ideal in terms of empowerment and connecting proposals with those they ultimately impact on.” These pilot testers felt the Equity Metrics were not responsive to those contextual constraints, and that it did not put enough emphasis on assessing change or reform that is initiated within broader systems and institutions.

Based on the feedback from the pilot testers, the Equity Working Group made revisions to the metrics, measurement scales, examples, and interview questions, and added introductory text describing uses of the metrics and the importance of community empowerment in health. Some of the more significant revisions included:

(1) The potential uses of the Equity Metrics were expanded, as noted below;
(2) A paragraph was added to the introduction to the metrics describing the Working Group’s understanding of why community empowerment and power redistribution are critical for advancing equity through HIA practice; and
(3) Two metrics were revised to increase HIA practitioners’ understanding of the context of how the proposal came about and the implications for equity.

The Equity Metrics may be used in a variety of ways by HIA practitioners, stakeholders, and decision-makers depending on the objectives as well as the time and other resources available to the user. The metrics can be used to:

(1) Comprehensively evaluate the degree to which an HIA successfully incorporated equity, using all of the metrics and the many sources of data available (including document review and interviews with other stakeholders);
(2) Employ the entire set of metrics as a self-reflective exercise, taking a more cursory approach with limited or no consideration of additional sources of data;
(3) Compare several HIAs using a subset of the metrics to evaluate how those HIAs addressed specific aspects of equity;
(4) Evaluate an HIA in a group discussion, using the metrics as a discussion guide;
(5) Advance equity through meaningful engagement and improved dialogue between stakeholders and decision-makers;
(6) Aid in planning an approach (at the start of an HIA, during screening and scoping) to advancing equity;
(7) Push HIA practitioners to achieve process and outcome objectives and to validate the multiple benefits of an HIA outside of solely impacting the decision outcomes; and
(8) Create benchmarks in legislation related to HIA and HiAP.

These metrics could be used to incorporate equity into a range of HIAs, from desktop to comprehensive. For example, a practitioner under time or resource constraints could refer to the metrics, or a subset thereof, to aid in planning an HIA approach that strategically uses limited resources to advance equity. Although these metrics were developed for utilization in HIA practice, they may also serve as a resource for more meaningful incorporation of equity into other types of impact assessment, such as Environmental Impact Assessment (EIA) or Regulatory Impact Analysis. For example, the process of developing an Environmental Impact Statement as part of an EIA presents opportunities for community engagement and responsiveness to community concerns in public comment periods, which can serve to surface and compel stronger consideration of equity impacts [12]. Conducting HIA to inform federal regulatory analysis and the development of regulations can also offer a framework for integrating environmental justice and equity considerations in policymaking [13].

Table 1 depicts an abbreviated version of the revised Equity Metrics. The full revised Equity Metrics can be found online [14].

**Table 1:** Equity Metrics for Health Impact Assessment Practice.

Metric	Description			Examples of High Scoring Activities/ Results	
**Outcome 1: The HIA Process and Product Focused on Equity**					
1.a	Proposal analyzed in the HIA was identified by and/or relevant to communities facing inequities			HIA practitioner asked community facing inequity what policy or plan they thought would have an impact on their health and proceeded with that as the HIA topic; practitioner asked community facing inequity what their main health concerns were, identified an HIA topic based on that, and gained community support for moving forward with the HIA; HIA practitioner analyzed the power, policy, and historical context of the decision to understand its relevance for equity	
1.b	The HIA scope—including goals, research questions, and methods—clearly addresses equity			At least one of the primary goals of the HIA is to assess equity impacts, whether or not the term equity is used; research questions call for focus on communities facing inequities	
1.c	Distribution of health and equity impacts across the population were analyzed (e.g., existing conditions, impacts on specific populations predicted) to address inequities; the HIA utilized community knowledge and experience as evidence			Quantitative assessment of disproportionate impacts (and potential cumulative impacts) on communities facing inequities included in the HIA; focus groups and/or surveys conducted in communities facing inequities	
1.d	Recommendations focus on impacts to communities facing inequities and are responsive to community concerns			Key recommendations focus on impacts to those facing inequities, not just on improving overall population health; recommendations reflect community priorities	
1.e	Findings and recommendations are disseminated in and by communities facing inequities using a range of culturally and linguistically appropriate media and platforms			Findings and recommendations translated into relevant languages and media formats (e.g., social media) and distributed; community leaders communicate findings on their own behalf to policy-makers and other community members	
1.f	Monitoring and evaluation (M & E) plan included clear goals to monitor equity impacts over time and an accountability mechanism (*i.e.*, accountability triggers, actions, and responsible parties) to address adverse impacts that may arise			During M & E, if negative equity impacts are found, decision-makers are responsible for implementing an improvement plan and reporting back to the community	
**Outcome 2: The HIA Process Built the Capacity and Ability of Communities Facing Health Inequities to Engage in Future HIA and in Decision-Making More Generally**					
2.a	Communities facing inequities lead or are meaningfully involved in each step of the HIA			For example, in the scoping stage this could include communities facing inequities having decision-making authority over the final Scope; in the assessment stage this could include utilizing community participatory methods	
2.b	As a result of the HIA, communities facing inequities have increased knowledge and awareness of decision-making processes, and attained greater capacity to influence decision-making processes, including ability to plan, organize, fundraise, and take action within the decision-making context			HIA process involved leadership training for members of communities facing inequities; HIA conducted in such a way as to increase understanding of action research as a tool for community change; community members have a better understanding of how to analyze the power, policy, and historical context of decisions	
**Outcome 3: The HIA Resulted in a Shift in Power Benefiting Communities Facing Inequities**					
3.a	Communities that face inequities have increased influence over decisions, policies, partnerships, institutions and systems that affect their lives			A shift in culture both within institutions and among communities about what is considered evidence (*i.e.*, community data or knowledge as “expert” and valid evidence); members of communities facing inequities get invited to have a seat at the decision-making table	
3.b	Government and institutions are more transparent, inclusive, responsive, and/or collaborative			Change in institutional design, such as Community Advisory Boards, new offices of Health Equity, or integration of equity into all missions	
**Outcome 4: The HIA Contributed to Changes that Reduced Health Inequities and Inequities in the Social and Environmental Determinants of Health**					
4.a	The HIA influenced the social and environmental determinants of health within the community and a decreased differential in these determinants between communities facing inequities and other communities			Determinants of health that were the focus of the HIA are improved in communities facing inequities at a faster rate than in the general population	
4.b (aspirational)	The HIA influenced physical, mental, and social health issues within the community and a decreased differential in these health outcomes between communities facing inequities and other communities			Health outcomes that were the focus of the HIA are improved in communities facing inequities at a faster rate than in the general population	

The wide variation in responses and feedback indicate that the conception and use of the metrics might vary by context and experience of the practitioner. For example, the experience of the practitioner in undertaking HIAs, the experience of the practitioner or evaluator with evaluation tools in general, the context in which HIA is being conducted, time or resource constraints, and/or the availability of shared vocabulary among HIA team members and stakeholders to describe equity-related concepts could all impact the use of the metrics. A recent national evaluation of HIAs pointed out that to bring equity considerations and the needs of vulnerable populations into the HIA process in more consistent and meaningful ways, practitioners could think through practical parameters such as the resources and team skills needed, the HIA timeline, and how shared expectations will be developed and managed [6].

Responses demonstrate that community engagement during all steps of HIA is not yet standard practice. They also reveal that “policy-making” and “community engagement” may be seen as two distinct, disconnected practices, though these same respondents did not view this paradigm as ideal. While community involvement does not necessarily lead to integration of equity in HIA, the involvement of communities facing inequities is likely to create pressure for improvements in institutions, systems, and belief systems that perpetuate inequities.

The subject of equity is complex and multifaceted; the authors acknowledge that summarizing equity into discrete component parts is a reductionist approach to a complicated topic. However, given that the vision for the Equity Metrics is that they should be able to be used by HIA practitioners from a range of backgrounds, experiences, and contexts, this approach is a necessity. The tradeoffs in the practicality of this approach outweigh the loss in nuance and complexity that a discussion of equity often demands. It is an attempt to connect theory to practice and provide a set of indicators to facilitate shared understanding in the field. These Equity Metrics are an effort to operationalize and embed the inclusion of equity through assessment of the processes and outcomes accomplished during and after the completion of HIAs. These actions are intended to shift power and contribute to advancing health equity and social justice. Outlining procedural approaches within HIA that can contribute to more equitable outcomes is a complement to existing theories of equity and community empowerment. We acknowledge that there may be additional considerations and other frameworks for including equity in HIA practice.

It is also necessary to acknowledge that the use of this tool does not guarantee health equity will be achieved. Achieving equity is a long-term proposition. The Equity Metrics provide a platform through which a more systematic consideration of health equity and the processes for incorporating equity into HIA can be accomplished. As such, this tool represents a starting point from which equity can be systematically and pragmatically advanced through the HIA process.

## 4. Conclusions

Equity is a core value of HIA, and this paper discusses the development of metrics that aim to guide and evaluate progress toward achieving equity in HIA practice. Over the course of a year, the metrics were developed as part of a consensus process among members of a working group affiliated with SOPHIA. The initial set of metrics was piloted and reviewed by HIA practitioners, and changes were made based on their feedback and recommendations. Development of this tool provided an enhanced understanding of the variations in paradigms regarding health equity and the role of community in HIA practice. Finally, development of the Equity Metrics confirmed the SOPHIA Equity Workgroup members’ initial assessment that there is a need for such a tool to help systematically guide HIA practitioners to include equity. These metrics are an attempt to catalyze and embolden HIA practitioners to deepen their commitment to equity.
